# A Culture-Based Study of Micromycetes Isolated from the Urban Nests of Grey Heron (*Ardea cinerea*) in SW Poland

**DOI:** 10.3390/ani12060676

**Published:** 2022-03-08

**Authors:** Rafał Ogórek, Justyna Borzęcka, Katarzyna Kłosińska, Agata Piecuch, Marcin Przymencki, Klaudia Litwiniak, Jakub Suchodolski

**Affiliations:** 1 Department of Mycology and Genetics, University of Wrocław, Przybyszewskiego Street 63/77, 51-148 Wrocław, Poland; justyna.borzecka@uwr.edu.pl (J.B.); 326428@uwr.edu.pl (K.K.); agata.piecuch@uwr.edu.pl (A.P.); jakub.suchodolski@uwr.edu.pl (J.S.); 2 Independent Researchers, Poleska Street 37/17, 51-148 Wrocław, Poland; marcin.przymencki@wp.pl (M.P.); kklitwiniak@gmail.com (K.L.)

**Keywords:** bird nests, urban area, fungal communities, zoopathogenic fungi

## Abstract

**Simple Summary:**

Fungi inhabiting bird nests may pose a serious threat to living organisms. Therefore, the main goal of the study was to identify cultivable fungi in the nest of grey heron (*Ardea cinerea*) located near the city centre of Wrocław (Poland). Overall, 10 different fungal species were obtained which were both cosmopolitan and potentially hazardous to humans, homoiothermous animals and plants. The greatest number of fungal species was obtained from the nest fragments with visible fungal growth, and the least from western conifer seed bugs (*Leptoglossus occidentalis*) inhabiting the nests. The damp chamber allowed isolation of *Aspergillus fumigatus*, *Penicillium coprophilum,* and *P. griseofulvum* as directly related to the occurrence of visible fungal growth on plant fragments of grey heron nests.

**Abstract:**

There are many positive relationships between micromycetes and birds: They can spread fungal spores, and fungi facilitate cavity woodpecker excavation by preparing and modifying excavation sites. In turn, bird nests are mainly a source of potentially zoopathogenic fungi. The Wrocław city centre hosts the biggest grey heron breeding colony in Poland with at least 240 breeding birds pairs. To assess the possible public health risks associated with bird nests, the goal of the present study was to identify cultivable fungi present in the nests of grey herons (*Ardea cinerea*) in Wrocław. Additionally, attempts were made to determine whether the obtained species of fungi may pose a potential threat to animal health. Fungi were cultured at 23 and 37 ± 0.5 °C, and identified based on phenotypic and genotypic traits. Moreover, during routine inspection, visible fungal growth in some of the nests was found. Overall, 10 different fungal species were obtained in the study (*Alternaria alternata*, *Aspergillus fumigatus*, *Botryotrichum piluliferum*, *Cladosporium cladosporioides*, *Epicoccum layuense*, *Mucor circinelloides*, *M.* *hiemalis*, *Penicillium atramentosum*, *P.* *coprophilum*, and *P.* *griseofulvum*). They are both cosmopolitan species and a source of potential threat to humans, homoiothermous animals and plants. The greatest number of fungal species was obtained from the nest fragments with visible fungal growth incubated at 23 °C, and the least from western conifer seed bugs (*Leptoglossus occidentalis*) inhabiting the nests. The species such as *A. fumigatus*, *P. coprophilum*, and *P.* *griseofulvum* can be directly related to the occurrence of visible fungal growth on plant fragments of grey heron’s nests.

## 1. Introduction

Birds-associated fungi can play either positive, neutral or negative roles in ecosystems [1,2,3,4,5,6,7]. Fungi allow excavator birds (such as woodpeckers), softening wood, while the birds may facilitate fungal spore dispersion [1,2]. Moreover, some bird species feast on fungi (54 bird species in 27 families), while others use fungal rhizomorphs in their nests construction (about 176 bird species in 37 families) [8]. Some examples of birds, which were described as fungal spore dispersal vectors, are chucao tapaculos (*Scelorchilus rubecula*) and black-throated huet-huets (*Pteroptochos tarnii*), which regularly consume mycorrhizal fungi and disperse viable spores via mycophagy [3]. Unfortunately, micromycetes associated with birds might constitute a potential threat not only to other animals but also to humans, leading to zoonotic diseases including cryptococcosis, or histoplasmosis [9,10]. Fungal infections in birds are common and might involve aspergillosis, candidosis, cryptococcosis, rhodotoruliasis, or mucormycosis [11,12]. The majority of fungal infections are caused by weakened immune system or poor nutrition. However, specific environmental conditions, such as high humidity, might also promote fungal growth. Therefore, such fungal infections have mainly opportunistic nature [4,5,6,7].

Fungi are also commonly found in bird droppings, which are good substrates for the growth of micromycetes due to, among other things, high concentration of nitrogenous bases. Fungi isolated from bird droppings belong to several genera like *Cryptococcus*, *Trichosporon*, *Aspergillus*, or *Penicillium*, which are common opportunistic pathogens of humans and common producers of mycotoxins [6,13]. Avian nests are also a potential source of phyto- and zoopathogenic fungi [14,15,16,17]. Studies of fungal species associated with bird nests concern both terrestrial and wetland bird nests; however, the data on this issue is still scarce. Birds’ body temperature (usually 39–43 °C) and specific conditions prevailing in nests make them a favorable environment for the development of fungi. The plant materials used to build the nests, bird faeces, animal remains, and keratin-rich substrates (feathers or hair), are suitable substrates for microbial growth. The fungal species diversity in the nests depends also on the nest location, which affects the source of nutrients [14,15]. Recent research has focused on keratinophilic fungi (inhibiting wetland bird nests due to the high amount of keratin and favorable environmental conditions, e.g., elevated humidity [15,16,17].

Grey heron (*Ardea cinerea*) is a large wading colonial bird of the heron family Ardeidae, native throughout temperate Europe, Asia, and partially Africa. In Poland it usually breeds on conifer trees [18,19]. While the colonies are often situated in forests or reedbeds [20], recently more of them have been described near human settlements [21]. Thus, the analysis of fungal diversity in the nests of grey heron (including species potentially hazardous to animals and humans) seems to be an important issue.

The goal of the present research was to provide the first report of microscopic fungi associated with the nests of grey heron, located in city centre of Wrocław (Poland), and to estimate whether the isolated species are potentially dangerous towards animals or humans.

## 2. Materials and Methods

### 2.1. Study Area

**Figure 1:**
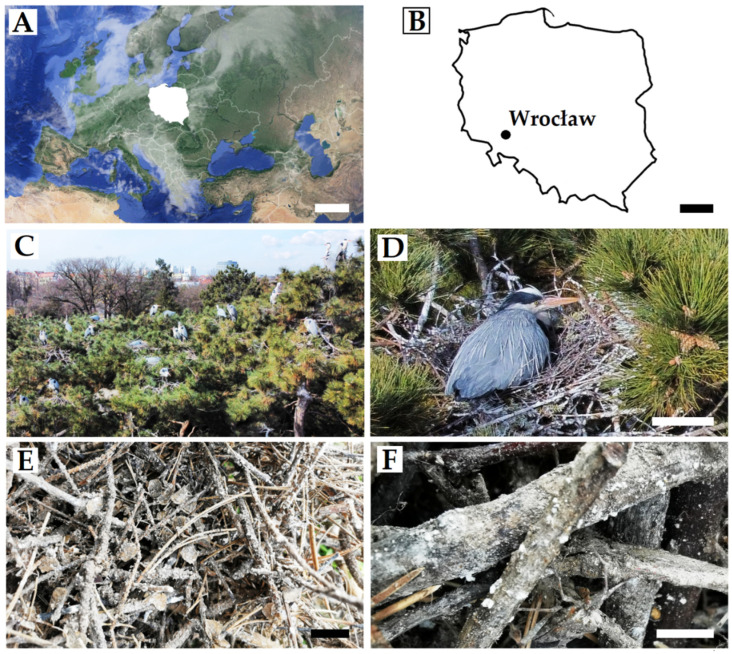
Geographic location of Poland (**A**) and the city of Wrocław (**B**). Grey heron breeding colony viewed from above (**C**), and close-up of female individuals in the nest (**D**). Nests with visible fungal growth (**E**,**F**). Scale bars: A = 500 km, B = 100 km, D = 20 cm, E = 1 cm, F = 5 mm.

The study was conducted in the city of Wrocław (Lower Silesia, Poland) (Figure 1). The location was chosen as the presence of the grey heron breeding colony in the city centre is a distinct nationwide phenomenon [21]. Despite its urban location, Beuch et al. detected 240 breeding pairs, describing it as the biggest grey heron colony in South-West (SW) Poland [22]. In Wrocław, grey herons nest in two specific locations: The zoo or partially in the biggest Lower Silesian park, Szczytnicki Park. In the zoo, grey herons use various deciduous trees and pines to form the foundations of their nests. In the Szczytnicki Park, however, grey herons settle using only black pines (*Pinus nigra*). In that area, grey herons pose a threat to passers-by and damage the historic/landmark trees growing there. In 2020, city authorities decided to drop all 144 nests in that sub-colony during non-breeding season. As a result, many nests were damaged and then fell off the trees. Only a few fallen nests were found intact.

### 2.2. Sample Collection

Field studies were conducted in 2021 between November 12 and November 14, during nest dropping in the colony. Biological material was sampled collectively using four out of five nests which fell on the ground intact. The samples consisted of the nests’ fragments with or without visible fungal growth. Additionally, in total 40 specimens of insects inhabiting the nests were sampled; these were western conifer seed bugs (*Leptoglossus occidentalis*), most likely wintering in the nests [23]. The material was collected in sterile ziplock bags using sterile pincers, was transferred to laboratory on the same day, and was stored at 5 ± 0.5 °C until analyzed (up to 5 days).

### 2.3. Isolation of Micromycetes from Samples Using Culture-Dependent Approach

#### 2.3.1. Procedure of Placing Biological Material on Petri Dishes

The nest material of plant origin was aseptically cut into 3 cm fragments and transferred onto PDA (Potato Dextrose Agar, Biomaxima, Poland) plates (5 fragments per plate) in five repetitions (*n* = 25 samples). Similarly, 5 specimens of *L. occidentalis* were placed on PDA plates in 5 repetitions (*n* = 25 samples).

The material was incubated in the darkness at 37 ± 0.5 °C for 3 to 5 days, and at 23 ± 0.5 °C for 4 to 14 days. Then, the grown fungal colonies were subcultured on PDA plates and incubated in darkness at 23 or 37 ± 0.5 °C. Fungi were purified by the single spore method and were put onto PDA slants for morphological and molecular identification.

#### 2.3.2. A Damp Chamber Procedure

For the identification of the dominant fungal species, nest fragments were additionally incubated in a damp chamber (made of sterile glass placed in Petri dishes) for 28 days at 23 ± 0.5 °C. The fungal overgrown material was impressed on the PDA substrate using sterile tweezers. Then, the grown fungal colonies were subcultured on PDA plates and incubated in darkness at 23 ± 0.5 °C. Fungi were purified by the single spore method and subcultured on PDA slants for morphological and molecular identification.

### 2.4. Fungal Identification

The obtained fungi were identified using classical phenotypic methods according to available monographs and articles [24,25,26,27,28,29,30,31], and a molecular analysis was performed. For this purpose, DNA was extracted from a 10-day-old culture on PDA using Bead-Beat Micro AX Gravity kit (A&A Biotechnology, Gdańsk, Poland) according to the manufacturer’s instructions. Fungal rDNA was amplified using ITS1 (5′-TCCGTAGGTGAACCTGCGG-3′) and ITS4 (5′-TCCTCCGCTTATTGATATGC-3′) primer pairs [24]. PCR was performed in a T100 Thermal Cycler (Bio-Rad, Berkeley, CA, USA) according to previously published protocol [32,33]. Briefly, after initial denaturation for 5 min at 94 °C, each cycle was comprised of a 30 s denaturation step at 94 °C, a 30 s annealing step at 55 °C, a 45 s extension step at 72 °C with a final extension step for 7 min at 72 °C by the end of 35 cycles. Amplification of fungal DNA was performed in a 50 μL reaction mixture using the 2 × PCR mixture containing a Taq polymerase (0.1 U × μL^−^^1^), dNTP mix (2 mM), MgCl_2_ (4 mM), 0.25 μM of each primer, and 45 ng of genomic fungal DNA [33]. The fungal internal transcribed spacer regions were verified by electrophoretic separation on a 1.2% agarose gel, purified using Clean-Up Kit (A&A Biotechnology, Gdańsk, Poland), and sequenced at Macrogen Europe (Amsterdam, The Netherlands, http://dna.macrogen.com/eng/ accessed on 20 January 2022).

### 2.5. Data Analyses

Raw sequence readings were analyzed using the BioEdit Sequence Alignment Editor (http://www.mbio.ncsu.edu/bioedit/bioedit.html accessed on 20 January 2022). Then, the obtained PCR products were compared with those deposited in the GenBank of the National Center for Biotechnology Information (NCBI, Bethesda, Rockville, MD, USA) using the BLAST algorithm (http://www.ncbi.nlm.nih.gov/ accessed on 20 January 2022), and submitted into this database (Table 1).

## 3. Results

All fungi isolated in the study were assigned to 13 distinct fungal isolates. The further phenotypical and molecular analyses of the isolates enabled their identification into two different phyla, five orders, and seven genera, including 10 different species, i.e., *Alternaria alternata*, *Epicoccum layuense* (Ascomycota, Pleosporales); *Aspergillus fumigatus*, *Penicillium atramentosum*, *Penicillium coprophilum*, *Penicillium griseofulvum* (Ascomycota, Eurotiales); *Botryotrichum piluliferum* (Ascomycota, Sordariales), *Cladosporium cladosporioides* (Ascomycota, Cladosporiales); *Mucor circinelloides*, *Mucor hiemalis* (Mucoromycota, Mucorales). Each group representative of the identified fungi received internal isolate numbers, namely UWR_210–UWR_218, UWR_262–UWR_264, and UWR_297. The ITS sequences obtained for the tested isolates ranged from 386 to 567 bp and were submitted to GenBank under accession numbers MW763150–MW763158, MZ391135–MZ391137, and OL657062. In the performed BLAST analysis, the E values were zero, the percentage of query cover or identity amounted to 100% or 99.48–100%, respectively (Table 1).

The micromycete species diversity depended on the incubation temperature. The most fungal species (*n* = 10) were isolated from the tested samples incubated at 23 ± 0.5 °C, and only two species at 37 ± 0.5 °C. The presence of all fungal species (*n* = 10) obtained at 23 ± 0.5 °C was found only on the nest fragments with visible fungal growth. In turn, eight species were isolated from the nest fragments without visible fungal growth and six from the surface of *L. occidentalis* (Table 2).

**Figure 2:**
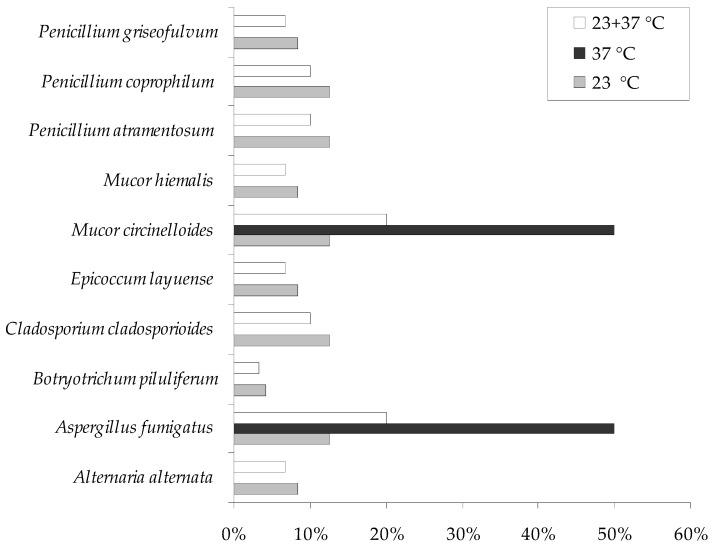
Percentage of each fungal species concerning the total isolated fungi from all studied biological materials of grey heron nests.

Overall, *A. fumigatus* and *M. circinelloides* were the most frequently isolated fungal species from the nests of grey heron (*A. cinerea*) and *L. occidentalis* inhibiting these nests; however, the incubation temperature affected the numbers of the cultured fungi. The above-mentioned species were isolated only at 37 ± 0.5 °C at the same level, *A. fumigatus*, *C. cladosporioides*, *M. circinelloides*, *P. atramentosum*, and *P. coprophilum* were frequently obtained at 23 ± 0.5 °C (Figure 2, Table 2).

**Figure 3:**
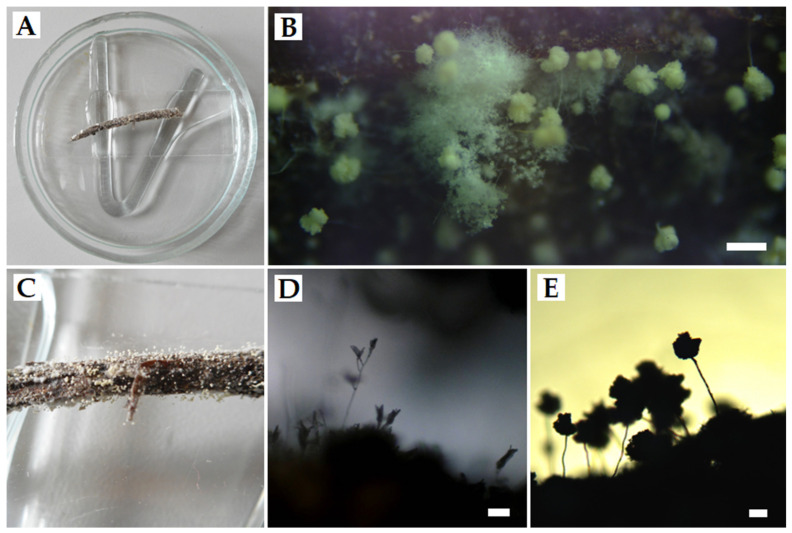
Nest fragments (**A**,**C**) after 28 days at 23 ± 0.5 °C incubation in a damp chamber and visible mycelium growth under the microscope (**B**). Close-up on the morphological structures produced by fungi of the genus *Penicillium* (**D**) and *Aspergillus* (**E**), which were identified by using phenotypic and molecular tests as *Penicillium coprophilum*, *Penicillium griseofulvum*, and *Aspergillus fumigatus*. Scale bars: B = 200 μm, D = 50 μm, E = 100 μm.

Analysis of the damp chamber-grown fungi showed that only three out of 10 species obtained in the study were closely related to the nest’s plant material—*P. coprophilum*, *P. griseofulvum*, and *A. fumigatus*. Those species dominated both on the fragments with and without visible fungal growth, which were subjected to a 28-day incubation procedure in excess moisture at 23 ± 0.5 °C (Figure 3, Table 1).

## 4. Discussion

The keratinolytic fungi have been the most studied fungal group inhabiting bird nests [15,16,34,35], despite the fact that nest composition should facilitate the growth of broader groups of fungi [6]. Nevertheless, the development of micromycetes on nests also depends on other factors, e.g., humidity. Nests of wetland birds (such as mute swan, *Cygnus olor*), located directly on water, are beneficial for the development of most fungi, including mainly hydrophilic species. On the other hand, grey heron nests located in trees favor the development of xerophilic, thermotolerant, and extremotolerant–extremophilic melanized fungi due to high sun exposure and consequently dryness [16,36]. The latter group of fungi respond to increased intensity of sunlight by overproduction of melanin [36]. Elevated melanin levels not only increases the survival of fungi in the environment but also directly interacts with the immune system, leading to reducing phagocytic cell effectiveness, binding effector or antifungal molecules, as well as modifying complement and antibody responses [37].

Hatchlings defecate in the nest, which increases salinity and alkalization of the nest lining, favoring alkalitolerant fungal species [16]. Probably the biology of the grey herons and the physicochemical properties of their nests affect only the small variety of cultivable fungi that inhabit them, which was shown in our research. However, the reason for obtaining a narrow spectrum of fungal species might have also originated in the use of culture-based methods for isolating fungi.

The traditional methods are more common and cheaper than high-throughput sequencing approaches. However, this approach has several disadvantages, including impossibility to detect unculturable elements [38,39,40,41,42]. On the other hand, our previous studies proved that slow-growing fungi (like Basidiomycota) are successfully isolated from the air using the PDA medium [43]. Nevertheless, the effectiveness of the culture-based analysis of fungal species depends, among other things, on the incubation temperature and type of culture medium [44,45]. Here, we used PDA and two incubation temperatures (23 and 37 ± 0.5 °C) to obtain a broader fungal species spectrum. The PDA medium is commonly used in mycological environmental analysis and is easy to prepare [43,46]. More importantly, this medium demonstrates comparable efficacy as Sabouraud agar, which is described as the most suitable for isolation of a large spectrum of fungal species from a variety of biological samples [47,48]. In turn, most fungi are grow at “room temperature” from 20 to 25 °C, but 37 °C is the temperature oscillating with respect to the body of mammals and thermophilic species [49].

The fungal species obtained in this study belong to seven genera: *Penicillium*, *Aspergillus*, *Mucor*, *Cladosporium*, *Botryotrichum*, *Epicoccum*, and *Alternaria*. Hubálek [14] analyzed avian nests from different habitats in former Czechoslovakia and Yugoslavia, and reported genera such as *Alternaria*, *Penicillium*, and *Mucor* (which corresponds to our findings), as well as *Arthroderma*, *Aphanoascus*, *Scopulariopsis*, *Chaetomium*, or *Fusarium* [14]. In Poland, most data originate from the work of Korniłłowicz-Kowalska et al., who deal with fungi associated with bird nests [15,16,17,50,51]. Overall, nests of wetland birds were described to contain fungi belonging to *Chrysosporium*, *Penicillium*, *Trichoderma*, *Trichophyton,* and *Microsporum* [15,16] or specifically *Aspergillus fumigatus* is most often isolated from these nests when the incubation temperature at 45 °C is used [17]. In turn, marsh harrier (*Circus aeruginosus*) nests are inhabited by *Aspergillus*, *Scopulariopsis*, *Chrysosporium*, and *Fusarium* genera [50], and the nests of Montagu’s harrier (*Circus pygargus*) are most often colonized by *Trichoderma*, *Aspergillus*, *Scopulariopsis*, and *Chrysosporium* spp. [51].

*Penicillium* spp. are conidiophore forming mold type of fungi which can be found in a variety of habitats. They produce a broad range of extracellular mycotoxins [31]. There were three different species of *Penicillium* genera found in the present research—*P. griseofulvum*, *P. coprophilum*, and *P. atramentosum*. The first of these (*P. griseofulvum*) is capable of producing several bioactive organic compounds such as patulin, penifulvin A, cyclopiazonic acid, roquefortine C, shikimic acid, 6-methylsalicylic acid, as well as a secondary metabolite called griseofulvin, known for its antifungal properties, used as a treatment of dermatophytoses [52,53]. Penifulvin A is claimed to be a novel and potent antiinsectan sesquiterpenoid. Roquefortine C is considered as a common metabolite produced by numerous *Penicillium* spp., such as *P. coprophilum,* also found in our study. Moreover, it is considered to be bacteriostatic against gram-positive bacteria, however, only in organisms which contain haemoproteins [54,55,56]. Two of the obtained species—*P. coprophilum* and *P. griseofulvum*—were found in the damp chambers. These fungi have some preferred nitrogen sources which are glutamine and ammonium, associated with nitrogen metabolite repression mechanisms. Because of that, the presence of nitrogen source in the growth environment has a huge impact on growth, physiological properties, and the activation of fungal differentiation. They are also able to utilize nitrates, glutamate, ammonium salts, glycine, acidic amino acids, or even more complex ones, like polyamines and proteins [57]. More importantly, *P. griseofulvum* causes avian penicillosis, which was found in a captive toucanet in Finland [58].

The next species found in the nests was *A. fumigatus*, a thermophilic mold (capable of growing even at 45 °C) commonly found in the nests of birds inhabiting wetlands [17]. It is capable of growing in pH 3.8–7.8, but at the same time has high requirements when it comes to the water presence, which oscillates from 0.9 to 0.95 [59]. *A. fumigatus* is considered as one of the most threatening opportunistic pathogens which causes pulmonary mycosis and pulmonary alveolitis in humans and birds. This specific species is responsible for 95% of aspergillosis cases in avian. Infection occurs through respiration of the spores. It is also capable of penetrating egg shells and membranes, leading to unhatched embryo death. *A. fumigatus* was one of the three species found in a damp chamber which might be connected to its capacity to create the mycelium and produce an extracellular enzyme—amylase. This phenomenon occurs under specific conditions, including nitrogen and carbon content in the medium, optimal pH around 6.00, quite common in heron nests as it was presented by Korniłłowicz-Kowalska et al. [15], and escalating from 5.99 to 7.76 [60]. Due to its enzymatic activity, this species is capable of biodegradation of plant originated materials which are commonly used as a nest building material [61].

In turn, *A. alternata* and *C. cladosporioides* rarely cause infections in humans and plants; however, they are highly allergenic and their spores trigger asthmatic reactions including seasonal allergy [62,63,64,65]. There are studies that have shown that the peak concentration of *A. alternata* and *C. cladosporioides* spores can be noted from June to September, while in the other months it can be equal to zero [66]. Due to the fact that grey heron nestlings enter the fledging stage of development about one or two months after being hatched around March or April, the spreading of the allergenic spores can be reinforced [67].

*Mucor* genus was represented in our study by two species: *M. hiemalis* and *M. circinelloides*, which were capable of growing at 37 °C. This trait fulfills the requirements of internal infections in mammals [68]. In fact, *M. hiemalis* and *M. circinelloides* can cause mucormycosis disease in immunocompromised humans and animals, which affects the nose, sinuses, eyes, and brain [69,70,71]. Other mucormycosis can spread to the lungs, stomach, intestines, and skin. In India, cases of diffuse thigh edema caused by *Mucor* spp. were described [71].

The last two species isolated in this study were *B. piluliferum* and *E. layuense*. The first of them is able to produce a numerous amount of mycotoxins, most notably oxisterigmatocystins E and F [72]. In turn, *E. layuense* is able to inhibit the growth of many phytopathogens, such as *Phaeomoniella chlamydospora* and *Fomitiporia mediterranea*, which infect grapevines [73].

## 5. Conclusions

Our study contributed to gaining new knowledge about micromycetes inhabiting the nests of grey heron (*Ardea cinerea*). We determined both the presence of cosmopolitan fungal species as well as pathogenic or potentially pathogenic fungal species in the nests to the grey heron species itself, and a source of mycological contamination in the environment. It was concluded that the visible fungal growth on plant fragments of the nests was caused by *Aspergillus fumigatus*, *Penicillium coprophilum,* or *Penicillium griseofulvum*. The present study is unique due to the specific environment in which the nests were found, namely in the city centre. Therefore, it is important to conduct further analyses of mycobiota nests in the future in order to maintain the biological safety of birds themselves and other mammals staying in the close neighborhood of grey heron nests, including humans.

## Figures and Tables

**Table 1:** BLAST analysis of rDNA ITS of fungi associated with grey heron (*Ardea cinerea*) nests or insects (*Leptoglossus occidentalis*) inhabiting the nests. All E values were equal to zero.

Fungi		Isolate	GenBank Accession No.	The Sequence Length (bp)	Identity with Sequence from GenBank			
					Query Cover, %	Identity, %	Accession	Isolate
Procedure of placing biological material on Petri dishes	*Alternaria alternata*	UWR_210	MW763150	475	100	100.00	MN615420	YZU 191238
	*Aspergillus fumigatus*	UWR_211	MW763151	505	100	100.00	MT597427	MEBP0074
	*Botryotrichum piluliferum*	UWR_212	MW763152	386	100	99.48	MH857131	CBS 131.53
	*Cladosporium cladosporioides*	UWR_213	MW763153	458	100	100.00	MT598826	T1
	*Epicoccum layuense*	UWR_214	MW763154	460	100	100.00	MT573479	17
	*Mucor circinelloides*	UWR_215	MW763155	567	100	100.00	MT603954	CMRC 573
	*Mucor hiemalis*	UWR_216	MW763156	523	100	100.00	MT366055	NG4
	*Penicillium atramentosum*	UWR_217	MW763157	446	100	100.00	MK281569	SCAU199
	*Penicillium coprophilum*	UWR_218	MW763158	512	100	100.00	MT410465	Mzz9
	*Penicillium griseofulvum*	UWR_262	MZ391135	490	100	100.00	MH006592	MPR1
A damp chamber procedure	*Aspergillus fumigatus*	UWR_297	OL657062	400	100	100.00	MT597433	MEBP0082
	*Penicillium coprophilum*	UWR_263	MZ391136	487	100	100.00	MK450685	CMV005G5
	*Penicillium griseofulvum*	UWR_264	MZ391137	440	100	100.00	KY069877	PPRI_22701

**Table 2:** Fungi isolated from grey heron nest fragments with/without fungal growth or from nest inhabiting insects (*Leptoglossus occidentalis*): x = the fungus was cultured from the samples.

Fungi	23 ± 0.5 °C			37 ± 0.5 °C		
	Nest with Visible Fungal Growth	Nest without Visible Fungal Growth	*L. occidentalis*	Nest with Visible Fungal Growth	Nest without Visible Fungal Growth	*L. occidentalis*
*Alternaria alternata*	x	x				
*Aspergillus fumigatus*	x	x	x	x	x	x
*Botryotrichum piluliferum*	x					
*Cladosporium ladosporioides*	x	x	x			
*Epicoccum layuense*	x	x				
*Mucor circinelloides*	x	x	x	x	x	x
*Mucor hiemalis*	x	x				
*Penicillium atramentosum*	x	x	x			
*Penicillium coprophilum*	x	x	x			
*Penicillium griseofulvum*	x		x			

## Data Availability

Not applicable.
